# The discovery of biological subphenotypes in ARDS: a novel approach to targeted medicine?

**DOI:** 10.1186/s40560-021-00528-w

**Published:** 2021-01-21

**Authors:** Karin Wildi, Samantha Livingstone, Chiara Palmieri, Gianluigi LiBassi, Jacky Suen, John Fraser

**Affiliations:** 1 The Critical Care Research Group, The Prince Charles Hospital, Clinical Sciences Building, Level 3, Chermside, Brisbane, QLD 4032 Australia; 2 Faculty of Medicine, The University of Queensland, Brisbane, Australia; 3 Cardiovascular Research Group, Basel, Switzerland; 4 School of Veterinary Science, the University of Queensland, Brisbane, Australia

**Keywords:** Acute respiratory distress syndrome (ARDS), Subphenotypes, Targeted treatment, Cluster analysis, Precision medicine, Predictive and prognostic enrichment, Biomarker

## Abstract

The acute respiratory distress syndrome (ARDS) is a severe lung disorder with a high morbidity and mortality which affects all age groups. Despite active research with intense, ongoing attempts in developing pharmacological agents to treat ARDS, its mortality rate remains unaltered high and treatment is still only supportive. Over the years, there have been many attempts to identify meaningful subgroups likely to react differently to treatment among the heterogenous ARDS population, most of them unsuccessful. Only recently, analysis of large ARDS cohorts from randomized controlled trials have identified the presence of distinct biological subphenotypes among ARDS patients: a hypoinflammatory (or uninflamed; named P1) and a hyperinflammatory (or reactive; named P2) subphenotype have been proposed and corroborated with existing retrospective data. The hyperinflammatory subphenotyope was clearly associated with shock state, metabolic acidosis, and worse clinical outcomes. Core features of the respective subphenotypes were identified consistently in all assessed cohorts, independently of the studied population, the geographical location, the study design, or the analysis method. Additionally and clinically even more relevant treatment efficacies, as assessed retrospectively, appeared to be highly dependent on the respective subphenotype. This discovery launches a promising new approach to targeted medicine in ARDS. Even though it is now widely accepted that each ARDS subphenotype has distinct functional, biological, and mechanistic differences, there are crucial gaps in our knowledge, hindering the translation to bedside application. First of all, the underlying driving biological factors are still largely unknown, and secondly, there is currently no option for fast and easy identification of ARDS subphenotypes. This narrative review aims to summarize the evidence in biological subphenotyping in ARDS and tries to point out the current issues that will need addressing before translation of biological subohenotypes into clinical practice will be possible.

## Introduction

Described first in 1967 [1], acute respiratory distress syndrome (ARDS) is an acute severe inflammation of both lungs caused by various etiologies, either by direct pulmonary or by indirect systemic injury [2–4]. Multiple and heterogenous causes are known to result in ARDS, which is pathophysiologically characterized by a profound damage to the alveolar-capillary barrier due to injury, resulting in overflooding of the alveolar space, causing an impossibility of an adequate gas exchange [2, 3].

**Fig. 1:**
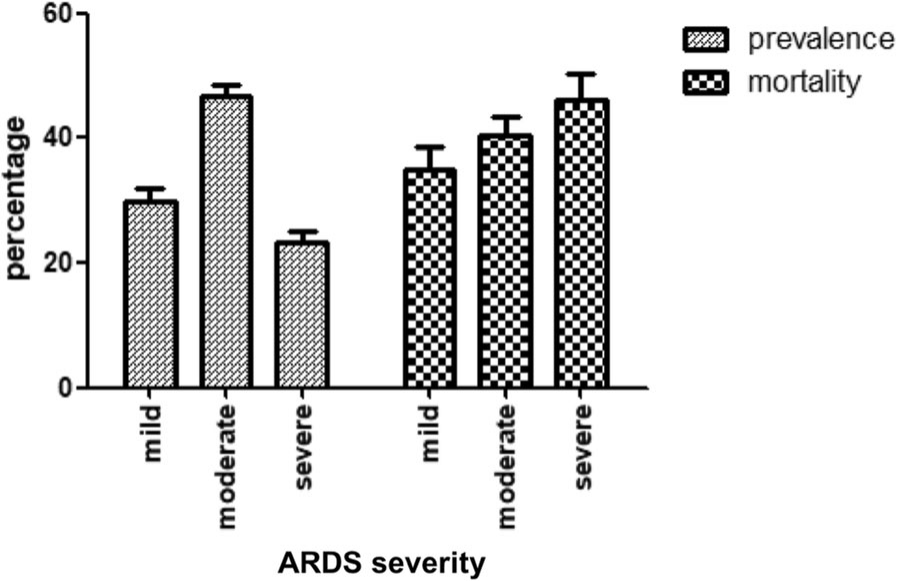
Mortality in ARDS according to the severity as defined by PaO_2_/FiO_2_-ratio

ARDS accounts for an average of 10.4% of all intensive care unit (ICU) admissions [5] with mortality ranging between 34.9% in mild cases to up to 46.1% in those with severe ARDS [5] as defined according to the Berlin definition [6] (Fig. 1). In survivors, quality of life is severely impaired [7], causing unsustainable human and economic burden. Considering the significant impact in health and economical terms, major research efforts have been conducted in the past 5 decades to more accurately characterize ARDS pathophysiology and to find an effective treatment. Unfortunately, research thus far has been largely unsuccessful in providing conclusive evidence of treatments that provide improved outcomes [8, 9], aside from supportive care to reduce ARDS mortality [8]. Consequently, regardless of the etiology or severity, ARDS patients are currently treated in a homogenous fashion [10].

However, it has to be emphasized that a good proportion of interventional studies evaluating treatment options in ARDS were conducted before the dogma change in mechanical ventilation toward lung-protective strategies [11]. Since it is now known that a non-protective ventilation strategy causes an additional inflammatory reaction [12, 13], a potential benefit of these applied treatments may therefore have been masked. Considering that the human ARDS population is highly heterogenous, it seems very likely that a uniform therapy non-selectively applied to all patients may further dilute any potential effect. These two factors could have been the main culprit of failure in previous studies.

This narrative review aims to provide an overview of the state of the current evidence in biological subphenotyping in ARDS regarding identified features, mortality rates, and different reaction to medical measures and treatment among patient subgroups. Additionally, we aim to identify important gaps in current knowledge that are to overcome in order to move forward in using biological subphenotyping in ARDS in future trials. This review focuses on biological subphenotyping only as this approach seems to be the most promising one for enrichment strategies in future ARDS trials.

## Approaches to subphenotyping in ARDS

**Fig. 2:**
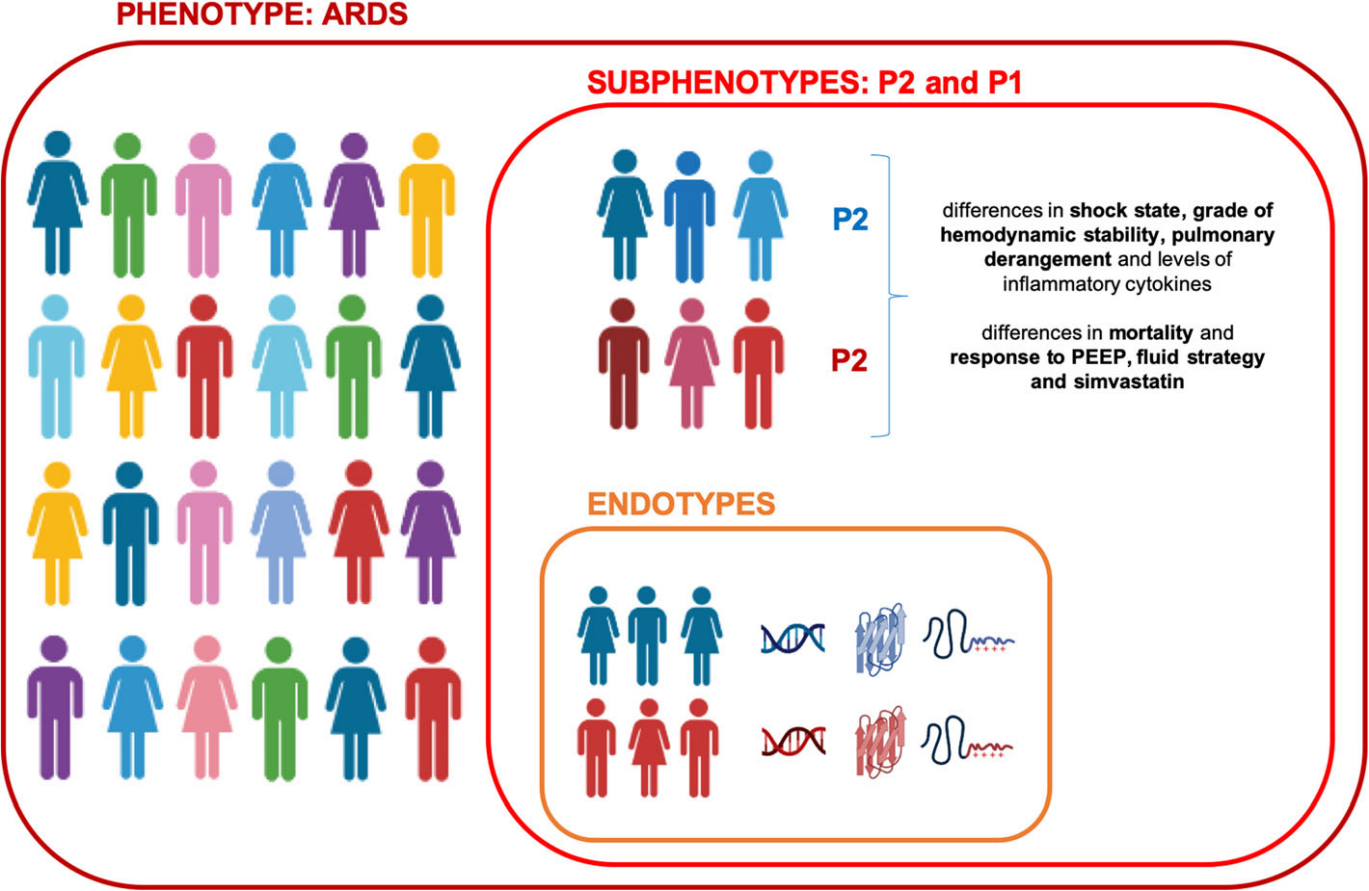
A phenotype denotes a group of patients that share a common syndrome, ARDS in this case. A subphenotype is a subset of patients within the phenotype that share specific features, such as clinical variables, outcomes, or responses to treatment or medical measures, that clearly differentiates this subgroup from others. An endotype is defined as a subgroup of patients within the subphenotype that have distinct biological mechanisms of the syndrome in common, such as gene expression and activated molecular pathways. For now, the definition of endotypes in ARDS is purely hypothetical as we know little about underlying biology

A subphenotype is defined as a subgroup among a disease entity that (a) is at highest risk for poor outcome (prognostic enrichment) or (b) shares similar underlying biological factors and/or a different reaction to medical measures (predictive enrichment) [14, 15] (Fig. 2). Enrichment strategies offer the potential to reduce heterogeneity and hence allow an approach to precision medicine by selecting the subgroup most likely to benefit [16].

Over the years, there have been several attempts to define subgroups among ARDS, either by differentiation according to the inflicting cause of ARDS as direct or indirect pulmonary injury [17, 18] or by confining trauma-related ARDS as this seemed to display different biological features [19, 20]. The Berlin definition [6] itself provides a prognostic enrichment as it divides the ARDS population into three severity subphenotypes according to the PaO2 to FiO2 ratio (PF ratio) with discriminative mortality rates [5]. From autopsy studies, we learnt about the presence of diffuse alveolar damage (DAD) [21–23], that was mainly found in moderate to severe ARDS [23] indicating a specific biological mechanism. Imaging studies reported that ARDS patients with diffuse radiological patterns displayed a higher mortality as compared to patients with focal patterns [24, 25]; these findings were associated with differences in pulmonary mechanics [26] but failed to result in different outcomes when applying a targeted ventilation approach [27].

All these subphenotyping attempts helped us clinicians to gain understanding about the complex syndrome of ARDS but were ultimately shown a weak or complete lack of evidence for a different treatment response or improved outcomes, most likely because underlying biological factors are yet to be completely understood. The novel concept of biological subphenotyes, two distinct subphenotypes, defined by specific functional and biological parameters, offers a novel and potentially more targeted approach to the very heterogenous population of ARDS. These biological subphenotypes were identified by latent class analysis (LCA), a novel statistical method for identifying unmeasured class membership among subjects, assuming that the data contains a certain number of unobserved groups (or classes). LCA uses an iterative algorithm by using mixture modeling, that identifies the best fit of number of classes between 1 and n for a data set and assigns each subject to a specific class [28, 29].

## The cornerstone of biological subphenotyping in ARDS

**Fig. 3:**
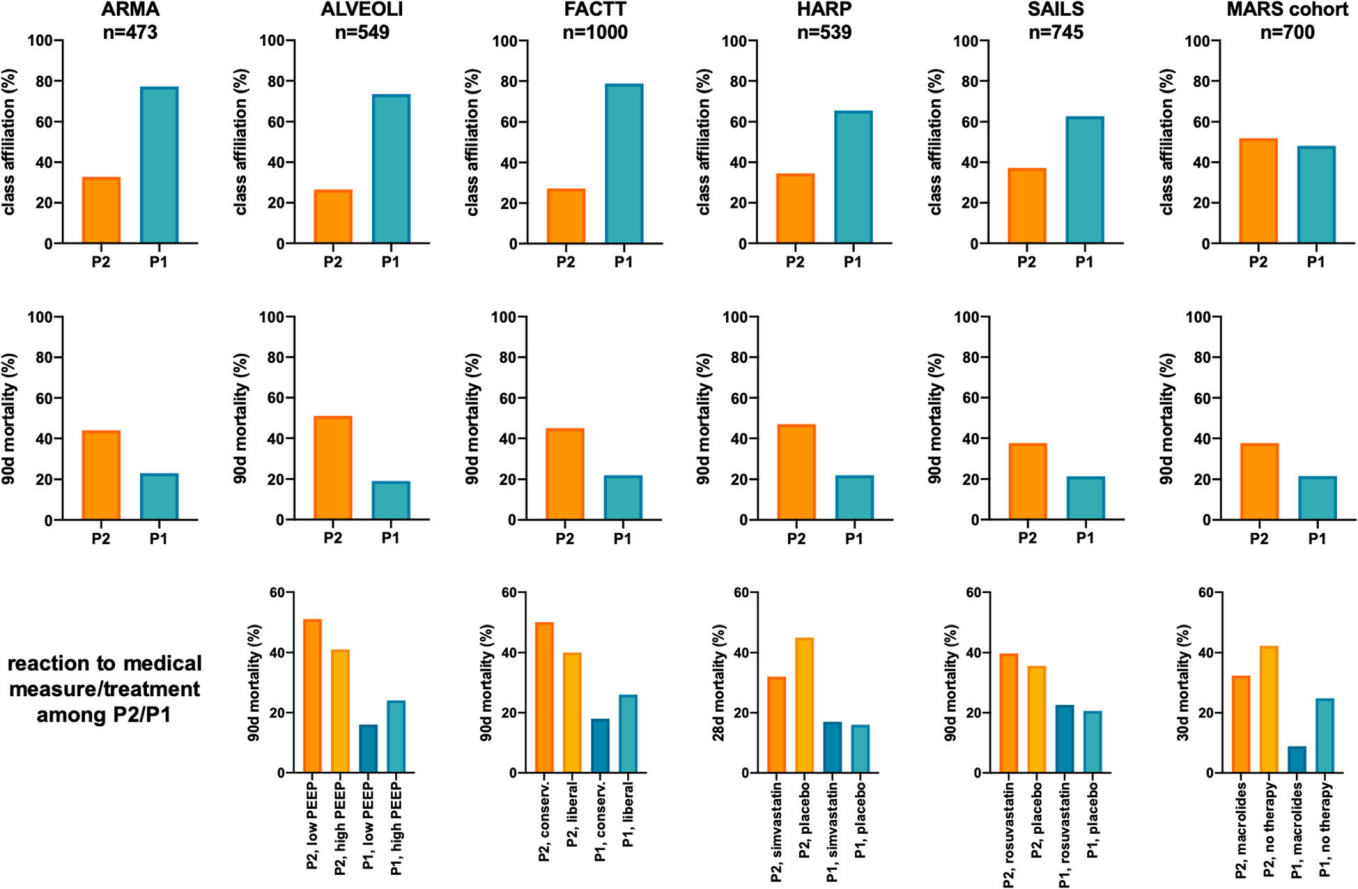
Class assignment to subphenotypes of ARDS, associated 90-day mortality and mortality according to different treatment. *ARMA* acute respiratory management of acute lung injury, *ALVEOLI* assessment of low tidal volume and elevated end-expiratory volume to obviate lung injury, *MARS* molecular diagnosis and risk stratification of sepsis, *FACTT* fluid and catheter treatment trial, *SAILS* statins for acutely injured lungs from sepsis, *HARP* hydroxymethylglutaryl-CoA reductase inhibition with simvastatin in acute lung injury to reduce pulmonary dysfunction, *PEEP* positive end-expiratory pressure, *P2* hyperinflammatory subphenotype, *P1* hypoinflammatory subphenotype

The origin of this new approach to ARDS was implemented in 2014 by Calfee et al. [30]. The group retrospectively analyzed two randomized controlled trials (RCT) from the National Heart Lung and Blood Institute (NHLBI) ARDS Network by LCA: the ARMA trial (Ventilation with Lower Tidal Volumes as Compared with Traditional Tidal Volumes for Acute Lung Injury and the Acute Respiratory Distress Syndrome) [11] that contributed 473 patients from the low tidal volume (VT) ventilation group (429 patients with high VT’s were excluded) and the ALVEOLI trial (Assessment of Low tidal Volume and elevated End-expiratory volume to Obviate Lung Injury) [31] which assessed different positive end expiratory pressure (PEEP) settings and contributed 549 patients to this analysis. Blood samples were taken at the time of randomization, < 36 h since fulfilling ARDS criteria. In the ARMA population as the derivation cohort, a two-class model was found to be the best fit and divided the population into a hyperinflammatory (named P2) and a hypoinflammatory subphenotype (named P1). One-third of patients were assigned to P2 (Fig. 3), with a significantly higher fraction of these patients being in shock. Dominant discriminating biomarkers were Interleukin (IL)-6 and -8, soluble tumor necrosis factor receptor 1 (sTNFR1), plasminogen activator inhibitor-1 (PAI-1), intercellular adhesion molecule-1 (ICAM-1), von Willebrand factor (vWF), bilirubin, bicarbonate, protein C (PC), PaCO_2_, platelets, albumin, and glucose. The clinical variables heart rate, minute ventilation, vasoactive use, plateau pressure, PEEP, and systolic blood pressure were shown to discriminate best between the subphenotypes (Table 1). Interestingly, neither the severity of ARDS as defined by the PaO_2_/FiO_2_ ratio (PF) [6], the severity of renal or hepatic failure, nor the extent of leukocytosis distinguished the two subphenotypes from each other. Risk factors for P2 were sepsis, pneumonia, and aspiration (in decreasing order), whereas in P1 it was pneumonia, sepsis, and aspiration. In comparison with the hypoinflammatory subphenotype, P2 displayed a higher 90-day mortality (44% vs. 23%, *p* = 0.006) (Fig. 3) and significantly less organ failure-free (9.8% vs. 16.8%; *p* < 0.001) as well as ventilator-free days (9.1% vs. 14.0%; *p* < 0.001). The same results were confirmed in the ALVEOLI cohort with remarkably similar characteristics of subphenotypes. Regarding outcome, the difference in 90-day mortality was even more pronounced with 51% and 19% in the P2 and P1 subgroups, respectively (*p* < 0.001) (Fig. 3).

**Table 1:** Characteristics of retrospectively assessed ARDS studies regarding ARDS subphenotypes: study design, analysis method, mortality, reaction to treatment, clinical variables, and biomarkers that differentiated best between subphenotypes of ARDS

	Country	Study design and analysis methodAnalysis method	90-day mortality	Evaluation of reaction among P2/P1 to	Clinical variables	Blood parameters
ARMA, *n* = 473 ALVEOLI, *n* = 549	USA (NHLBI)	RCT LCA	ARMA P2 44% P1 23% ALVEOLI P2 51% P1 22%	ALVEOLI: application of PEEP	Circulatory: heart rate, BPm, vasoactive use Respiratory: minute ventilation, Pplat, PEEP	Inflammation: IL-6, IL-8, sTNFR-1, CRP, WCC Coagulation: PAI-1, protein C, platelets Endothelial: Ang-2, ICAM-1, vWF Others: bilirubin, bicarbonate, PaCO_2_, albumin, glucose
MARS, *n* = 700	NL	Observational cohort Clustering methods	P2 37.7% P1 21.6%	Macrolide Antibiotics	None	Inflammation: IL-6, IL-8, IL-10, IFN-y Enothelial: Ang-1, Ang-2 Coagulation: PAI-1, antithrombin
FACTT, *n* = 1000	USA (NHLBI)	RCT LCA	P2 45% P1 22%	Fluid strategy	Circulatory: heart rate, BPs, vasoactive use Respiratory: minute ventilation, airway pressure, PEEP	Inflammation: IL-6, IL-8, TNFR-1, IFN-y Coagulation: PAI-1, protein C, platelets Endothelial: Ang-2, vWF Lung epithelial: RAGE Others: bilirubin, bicarbonate, creatinine, PaCO_2_, albumin, glucose, glucose
SAILS, *n* = 745	USA (NHLBI)	RCT LCA	P2 37.6% P1 21.4%	Rosuvastatin	Circulatory: heart rate, BPs, vasoactive use Respiratory: minute ventilation, respiratory rate, pulmonary risk factors others: urinary output	Inflammation: IL-6, IL-8, sTNFR-1, WCC Coagulation: protein C, platelets, PAI-1, platelets Endothelial: ICAM-1 others: bilirubin, bicarbonate, creatinine, PaCO_2_, albumin, glucose
HARP, *n* = 539	UK/IR	RCT LCA	P2 47% P1 22%	Simvastatin	Circulatory: vasoactive use Pulmonary: PF ratio	Inflammation: IL-6, sTNFR-1 Coagulation: platelets Others: creatinine, bilirubin
Kitsios et al., *n* = 212	USA (NHLBI)	Observational cohort LCA	ARDS P2 44% P1 22% ARFA P2 53% P1 18%	None	Circulatory: BPs, heart rate Pulmonary: PEEP, Pplat, PF ratio, respiratory rate, PF ratio Others: temperature	Inflammation: IL-6, IL-8, IL-10, TNFR-1, WCC, PCT Coagulation: protein C, platelets Endothelial: ICAM-1, Ang-2 Lung epithelial: RAGE Others: creatinine, PaCO_2_, ST-2; fractalkine, pentraxin3, pH art

*ARMA* Acute Respiratory Management of Acute lung injury, *ALVEOLI* assessment of low tidal volume and elevated end-expiratory volume to obviate lung injury, *MARS* Molecular diagnosis and risk stratification of sepsis, *FACTT* Fluid and Catheter Treatment Trial, *SAILS* statins for acutely injured lungs from sepsis, *HARP* hydroxymethylglutaryl-CoA reductase inhibition with simvastatin in acute lung injury to reduce pulmonary dysfunction, *RCT* randomized controlled trial, *LCA* latent cluster analysis, *P2* hyperinflammatory subphenotype, *P1* hypoinflammatory subpenotype, *ARFA* at risk for ARDS, *BPm* mean blood pressure, *BPs* systolic blood pressure, *Pplat* plateau pressure, *PEEP* positive end-exspiratory pressure, *IL* interleukin, *sTNFR*-*1* soluable tumor necrosis factor receptor-1, *CRP* C-reactive protein, *WCC* white cell count, *PAI*-*1* plasminogen inhibitor-1, *Ang*-*1*/-*2* angiopoetin-1/-2, *ICAM*-*1* intracellular adhesion molecule-1, *vWF* von Willebrand factor, *PaCO*_2_ arterial CO_2_ partial pressure, *IFN*-*γ* interferon gamma, *RAGE* receptor for advanced glycation end-products, *PF ratio* PaO_2_/FiO_2_ ratio; *ST*-*2* suppression of tumorigenicity, *PCT* procalcitonin

The FACTT trial (Fluid and Catheter Treatment Trial) [32] was another NHLBI-promoted study, randomizing ARDS patients in a two-by-two factorial design into 2 study arms for comparing fluid-liberal versus fluid-restrictive volume strategy and monitoring with pulmonary artery catheter versus central venous catheter, respectively. No difference in mortality at 60 days was found with either intervention, but significantly more ventilator-free days occurred in patients randomized to the fluid-conservative group. Famous et al. [33] analyzed the 1000 included patients retrospectively and found again that a 2-class model was the best fit, with 27.2% of patients assigned to P2 and 72.8% to P1 (Fig. 3). Best discriminating biomarkers in this cohort were once again IL-8 and -6, PAI-1, vWF, bilirubin, bicarbonate, PC, PaCO_2_, platelets, albumin, but also TNFr-1, angiopoetin-2 (Ang-2), receptor for advanced glycation endproducts (RAGE), and creatinine. In terms of clinical variables, heart rate, minute ventilation, airway pressures, vasoactive use, PEEP, and systolic blood pressure distinguished most accurately between subphenotypes. These findings were later validated in the ARMA and ALVEOLI cohorts. Again, 90-day mortality was significantly higher in P2 than P1 with 45% and 22% respectively (Fig. 3). Sepsis was a risk factor for ARDS in P2 subphenotype in 53%, whereas trauma, aspiration, and pneumonia were more likely in P1.

## The proof of stable class assignment over time

In order to understand the pathogenesis of subphenotypes in ARDS, knowledge about stability of subphenotypes over time is crucial. This task was accomplished by Delucchi et al. [34] in the ARMA and ALVEOLI cohorts through analysis on day 0 and 3 with a latent transition model. Authors founds evidence for stable classes over the first 3 days with the majority of patients being in the same class at day 0 and day 3 respectively. Only 5% of patients had a change in class (more frequently from P1 to P2), whereas the clinical outcome was associated with the later class. These important findings add further evidence to the hypothesis that there are fundamental biological and clinically relevant differences in subphenotypes in ARDS, concluding that these are not manifestations of different stages of the same disease as the subphenotype patterns are not affected by the measurement time point.

## The validation of ARDS subphenotypes in two European cohorts

Bos et al. 2017 [35] chose hierarchical clustering as an approach for the analysis of the MARS cohort (Molecular Diagnosis and Risk Stratification of Sepsis), a biobank initiative in sepsis, conducted in 2 ICU’s in the Netherlands between 2011 and 2013. In this analysis, ARDS was clustered according to biomarkers only and then associated with outcome. A total of 700 patients were available for analysis, divided in 454 for the training and 246 patients for the validation group. A reactive and an uninflamed subphenotype was defined with an ICU mortality of 36.4% and 15.6% accordingly (Fig. 3). The reactive subphenotype was characterized by higher Acute Physiology and Chronic Health Evaluation scores (APACHE), more severe multi-organ failure and indirect causes of ARDS. The dominant discriminant biomarkers between the two subphenotypes were IL-6, -8, -10, interferon-gamma (IFNγ), Ang-1/-2, and PAI-1 (Table 1). A 5-factor model, consisting of IL-6, IFN-γ, Ang-1/2, and PAI-1, provided an area under the curve (AUC) of 0.98 (95%CI 0.97 to 0.99) for discrimination between subphenotypes. Even though the class assignment to the reactive subpenotype was twice as high than reported previously [30, 33, 34], mortality and defining biomarkers of the two subphenotypes were quite comparable, suggesting that a similar cluster exists across all these cohorts. The difference in prevalence may be explained by a selection bias in RCT`s or underlying biological factors.

The HARP-2 trial (hydroxymethylglutaryl-CoA (HMG-CoA) reductase inhibition with simvastatin in acute lung injury to reduce pulmonary dysfunction) [36] was a multicenter RCT of simvastatin daily versus placebo in ARDS, conducted in 40 ICUs in the UK and Ireland over 4 years, randomizing 540 patients. No significant difference was detected between the study groups regarding 28-day mortality or number of ventilator-free days in the original study. Yet, in a secondary analysis by Calfee et al. [37], a 2-class model was again the best fit: 65% of patients were assigned to P1 and 35% to P2 subphenotype (Fig. 3). The best discriminating variables were sTNFR-1, creatinine, IL-6, bilirubin, platelets, vasoactive use, and the PF-ratio (Table 1). P2 experienced less ventilator-free days (2 vs. 18 days), fewer non-pulmonary organ failure-free days (15 vs. 27 days), and a higher 90-day mortality (47 vs. 22%; all *p* < 0.001) than the P1 subphenotype (Fig. 3). The most common ARDS risk factors sepsis, pneumonia, and aspiration were highly prevalent in both groups.

## Findings from the SAILS cohort

As another 3-HMG-CoA-reductase inhibitor, rosuvastatin was tested versus placebo for its efficacy in ARDS in the SAILS study (Statins for Acutely Injured Lungs from Sepsis). In this NHLBI ARDS Network trial in infection-associated ARDS [38], including 745 patients between 2010 and 2013, no difference in mortality was found between the groups. The SAILS cohort was retrospectively analyzed [39], using LCA for clinical variables and biomarkers, and consistently, a two-class model was found to be the best fit. Further, 227 patients (37%) were assigned to P2 and 448 patients (63%) to the P1 subphenotype. The 60-day mortality of 36.5% and the 90-day mortality of 37.6% was significantly higher in the P2 group than in the patient group assigned to P1 (20.9% and 21.4% respectively, all comparison *p* < 0.0001) (Fig. 3). Furthermore, the P2 group experienced fewer ventilator-free days (15 vs. 23 days; *p* < 0.0001). The class defining features were consistent with the previous analysis of the three NHLBI cohorts (ARMA, ALVEOLI, FACTT): IL-6 and -8, sTNFR-1, ICAM-1, PAI-1, PC, PaCO_2_, platelets, bicarbonate, albumin, bilirubin, creatinine, systolic blood pressure, heart and respiratory rate, vasoactive use, minute ventilation (Table 1). In addition, the P2 group had a higher white cell count, lower urinary output, and more pulmonary risk factors for ARDS. Also consistently with the results from the NHLBI ARDS Network datasets, respiratory variables including the PF-ratio performed poorly in discriminating between classes. The prominence of biomarkers as class defining variables suggests that subphenotypes may primarily be governed by basic biological factors. The authors concluded that these 4 NHLBI ARDS network datasets consistently reveal the same subphenotypes. This underlines their contemporaneous relevance despite changing demographical patterns and clinical practice in ARDS [40]. The replication of the results in ARDS cohorts from the UK/Ireland [37] and the Netherlands [35] proves the robustness and generalizability of the subphenotype model intercontinentally.

## Different reaction to medical measures among the subphenotypes

By analyzing the ALVEOLI cohort, a significant interaction between class assignment and PEEP settings as medical intervention was noted [30]. The P2 subphenotype displayed a 90-day mortality rate of 51% with low PEEP and of 40% with high PEEP, whereas in P1 the mortality rate of the two PEEP settings was 16% and 24% (*p* = 0.049) (Fig. 3). An even stronger interaction was seen between subphenotype and PEEP strategy regarding ventilator-free and organ failure-free days, where the P2 with a high-PEEP strategy showed significantly lower numbers for both outcomes. The authors concluded that the significant differences in natural histories, clinical, and biological characteristics as well as outcomes and response to treatment among the two different ARDS subphenotypes are characteristic requirements that have to be fulfilled to define a subphenotype.

In contra distinction to the findings of the original FACTT cohort as outlined above, differences in 90-day mortality relating to the applied fluid strategy were identified in the two identified subphenotypes [33]: P1 had a higher mortality with liberal compared to conservative fluid management (26 vs. 18%) and P2 was shown a higher mortality with conservative compared to liberal fluid management (50 vs. 40%) (Fig. 3).

De Simonis et al. [41] analyzed the MARS cohort regarding a treatment effect of macrolide antibiotics on subphenotypes using propensity-score (PS) matching. Then, 715 patients without macrolides were 3/1-matched to 158 patients with macrolide treatment (97% erythromycin). Most patients were treated within 5 days of ARDS diagnosis for a total of 3 days. Overall, patients with macrolides had an odds ratio for mortality of 0.64 (*p* = 0.03); this remained significant after PS-matching. The mortality at 30 days was specifically lower in non-pulmonary ARDS after PS-matching and in the P1 subphenotype before and after PS-matching (Fig. 3). The authors concluded that the effect was most probably mediated through a reduction in cytokines and an effect on neutrophil granulocytes.

Although the HARP-2 trial showed no difference in adjudicated outcomes, the secondary analysis [37] identified a different response to simvastatin when splitting the cohort into subphenotypes: P2 patients treated with simvastatin had a lower 28-day mortality with 32% (27/84) vs. 45% (46/102) (*p* = 0.008) in the placebo group. This was not observed in P1 where the 28-day mortality was 17% in the treatment group and 16% in the placebo group (*p* = ns) (Fig. 3).

Interestingly, in the SAILS cohort [39] as opposed to HARP-2, there was no difference in all three outcome measures in the P2 subphenotype regarding treatment with rosuvastatin (Fig. 3). While SAILS assessed infection-related ARDS, HARP-2 included a much wider variety of ARDS risk factors, therefore the identified subphenotypes may differ between the two cohorts. In addition, it was postulated that the use of a different 3-HMG-reductase inhibitor might explain the difference in outcome: while simvastatin is a lipophilic molecule with some clinical evidence in lung injury [42], rosuvastatin is hydrophilic with known different influence on plasma levels of inflammation markers [43]. Therefore, the use of a hydrophilic statin may be responsible for the negative results in the retrospective analysis of the SAILS dataset.

## Subphenotypes in patients at risk for ARDS?

In a recent publication [44], LCA was applied to baseline clinical variables and biomarkers in patients with ARDS as well as in patients at risk for ARDS (ARFA) but not entirely fulfilling the diagnostic criteria. Interestingly, a two-class model provided the best fit in both patient groups, whereas 38% (39/104) of ARDS and 28% (30/108) of ARFA patients were assigned to the hyperinflammatory subphenotype. The differentiating variables between the subphenotypes were comparable to the ones previously reported (Table 1). Both, hyperinflammatory ARDS and ARFA, were shown a higher 90-day mortality than hypoinflammatory subphenotypes (44% vs. 33% and 53% vs. 18%) but statistical significance was only reached in ARFA. These findings suggest that likely the extent of subphenotypes is not restricted to fully developed ARDS but are already present in preliminary stages due to similar driving factors.

## The gaps in the current knowledge

All these outlined results underline that most likely similar subphenotypes are observed among ARMA, ALVEOLI, FACTT, and SAILS, as well as among HARP-2 and the MARS cohort, which highlights the generalizability of subphenotypes among varying ARDS populations. Although these recent developments in ARDS research are very exciting and promising, there are still major challenges to overcome.

First, the underlying driving biological factors are still largely unknown. The key to a more thoroughly understanding may lay in omics data generation and application [45, 46]. Analyzing leukocyte expression profiles in the MARS cohort [47] was the first attempt to more fully understand molecular pathways in subpenotypes in ARDS by comparing differential gene expression that might be indicative of pathophysiologic changes within the subphenotype. The respective subphenotype was identified by the 5-factor-model [35] as previously derived in the MARS dataset [35]. Among 210 patients, 82 (38%) were assigned to the uninflamed (P1) and 128 (62%) patients to the reactive/hyperinflammatory subphenotype (P2). These were compared to 547 patients with sepsis but no ARDS and 42 healthy age-matched controls. Twenty-nine percent (3332/11443) of genes were significantly differently expressed between subphenotypes. In P2, 7 of 8 genes previously positively associated with ARDS [48], were shown to be upregulated, with pathways of oxidative phosphorylation (indicative of mitochondrial dysfunction) as well as cholesterol metabolism and the innate immune system being the most enriched ones. Fifty percent of genes that were previously found to be negatively associated with ARDS were upregulated in P1. Specifically, pathways coordinating cell proliferation and differentiation, motility and survival as well as the adaptive immune system were enriched in P1. Interestingly, sepsis patients without ARDS were most similar to P1 subphenotypes expression. While these results provide a glimpse to potential revelation of different underlying biological factors, we are still far away from an in-depth understanding. Previous studies with whole blood gene expression studies failed to prove a consistent gene signature for ARDS patients [49], assumingly because of a mixture in ARDS subphenotypes. In the near future, bioinformatic approaches like genomics, proteomics, transcriptomics and metabolomics will enhance our understanding of driving factors on a molecular level.

The second significant gap inhibiting current clinical application lies in the lack of an reliable and easy to use biomarker for differentiation between subphenotypes at the bedside. A first attempt at solving this hindrance was recently published [50]. Machine learning algorithms were applied to 3 cohorts from the NHLBI ARDS Network (ARMA, ALVEOLI, FACTT) incorporating 2200 patients, to select the six most important classifier variables for development of nested logistic regression models. The logistic regression models with the highest predictive accuracy were then evaluated in the validation cohort (SAILS; *n* = 715). The most important classifier factors were IL-8, -6, PC, sTNFR-1, bicarbonate and vasoactive use. A 4-variable model, incorporating IL-8, bicarbonate, PC, and vasoactive use, resulted in an AUC of 0.95 (95%CI 0.93–0.96) and performed best as compared with the LCA classification as the gold standard. Similar to the LCA-derived subphenotypes, P2 derived from the classifier model was shown to have a higher 90-day mortality than P1 (39% vs. 23%, *p* < 0.0001) and fewer ventilator-free days. However promising, so far there is no point-of-care test to identify subphenotypes in the clinical setting.

Third, even if there was a bedside test to select a specific subphenotype, the resulting clinical relevance is yet to be determined since the retrospective data have shown conflicting results regarding a treatment benefit in one specific ARDS subphenotype [37, 39, 41].

Fourth, the extent and clinical relevance of subphenotypes beyond ARDS has to be determined. The results by Kitsios et al. [44] are promising as we start to suspect that the true extent of subphenotypes is much larger than assumed and not only related to hypo- or hyperinflammatory states and maybe not even limited to lung failure [51, 52]. Validation in a larger cohort of patients with heterogenous risk factors for ARDS and a model to predicate its stability is needed.

Within the coming years, very likely we will have a more in-depth understanding of underlying disease mechanisms. The key to successfully translate this knowledge will lay in predictive enrichment [14, 53], meaning that reducing heterogeneity and thereby improving trial efficiency by refining patient selection and choosing patients more likely to respond to drug treatment will facilitate personalized medicine in this field and increase absolute and relative effects, as it has been shown previously [54–58].

## Conclusion

The clinical and biological heterogeneity of the ARDS population continues to gain acceptance in the clinical community, and might explain the five decades of ARDS research without treatment success. Subphenotyping provides a new promising approach for therapeutic development through the concept of predictive and prognostic enrichment, potentially resulting in a more targeted treatment. Nevertheless, there are crucial gaps yet to overcome, namely a more in-depth understanding of the underlying driving biological factors and a reliable biomarker for early differentiation between subphenotypes at the bedside. Once these hindrances have been resolved, subphenotyping will most likely be the key factor in all future pursuits in ARDS treatment.

## Data Availability

Not applicable.

## Abbreviations

ALVEOLI: Assessment of Low tidal Volume and elevated End-expiratory volume to Obviate Lung Injury
Ang-1/-2: Angiopoetin-1/-2
APACHE: Acute Physiology and Chronic Health Evaluation score
ARDS: Acute respiratory distress syndrome
ARFA: At risk for ARDS
ARMA: Ventilation with Lower Tidal Volumes as Compared with Traditional Tidal Volumes for Acute Lung Injury and the Acute Respiratory Distress Syndrome
AUC: Area under the curve
DAD: Diffuse alveolar damage
FACTT: Fluid and Catheter Treatment Trial
HARP-2: Hydroxymethylglutaryl-CoA Reductase inhibition with simvastatin in acute lung injury to reduce Pulmonary dysfunction
HMG-CoA: Hydroxymethylglutaryl-CoA
ICAM-1: Intercellular adhesion molecule-1
ICU: Intensive Care Unit
IFNy: Interferon-y
IL: Interleukin
LCA: Latent cluster analysis
MARS: Molecular Diagnosis and Risk Stratification of Sepsis
NHLBI: National Heart Lung and Blood Institute
PaCO_2_: Arterial CO_2_ partial pressure
PaO_2_: Arterial O_2_ partial pressure
PAI-1: Plasminogen activator inhibitor-1
PC: Protein C
PEEP: Positive end-expiratory pressure
PF ratio: PaO_2_/FiO_2_ ratio
P1: Uninflamed or hypoinflammatory subphenotype
P2: Hyperinflammatory or reactive subphenotype
PS: Propensity score
RAGE: Receptor for advanced glycation endproducts
RCT: Randomized controlled trial
SAILS: Statins for Acutely Injured Lungs from Sepsis
(s)TNFr-1: (Soluble) tumor necrosis factor receptor-1
VT: Tidal volume
vWF: Von Willebrand factor
