# Correction to: The discovery of biological subphenotypes in ARDS: a novel approach to targeted medicine?

**DOI:** 10.1186/s40560-021-00534-y

**Published:** 2021-02-25

**Authors:** Karin Wildi, Samantha Livingstone, Chiara Palmieri, Gianluigi LiBassi, Jacky Suen, John Fraser

**Affiliations:** 1 The Critical Care Research Group, The Prince Charles Hospital, Clinical Sciences Building, Level 3, Chermside, Brisbane, QLD 4032 Australia; 2 Faculty of Medicine, The University of Queensland, Brisbane, Australia; 3 Cardiovascular Research Group, Basel, Switzerland; 4 School of Veterinary Science, the University of Queensland, Brisbane, Australia

**Correction to: J Intensive Care 9, 14 (2021)**

**https://doi.org/10.1186/s40560-021-00528-w**

Following the publication of the original article [1], the authors identified that a figure is missing, and should be added as new Fig. 2. The original Fig. 2 are changed to Fig. 3 now.

**Fig. 2:**
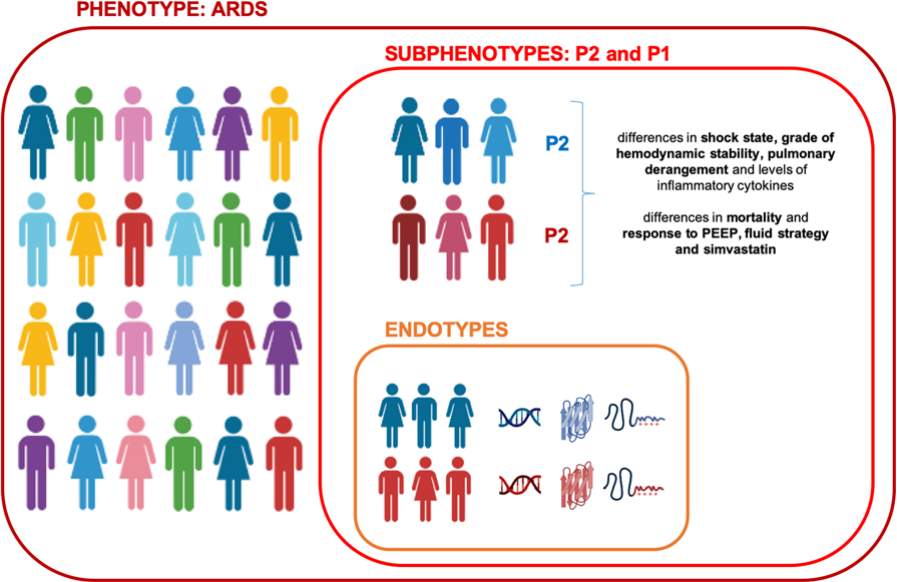
Infographic: visual explanation of the concepts of phenotypes, subphenotypes, and endotypes in ARDS. A phenotype denotes a group of patients that share a common syndrome, ARDS in this case. A subphenotype is a subset of patients within the phenotype that share specific features, such as clinical variables, outcomes, or responses to treatment or medical measures, that clearly differentiates this subgroup from others. An endotype is defined as a subgroup of patients within the subphenotype that have distinct biological mechanisms of the syndrome in common, such as gene expression and activated molecular pathways. For now, the definittion of endotypes in ARDS is purely hypothetical as we know little about underlying biology.

The new Fig. 2 has been included in this correction, and the original article has been corrected.
